# From Antipsychotic to Antitumor Agent: Cariprazine Suppresses Glioblastoma via D2/D3-ARRB2 Axis Modulation

**DOI:** 10.3390/ph19060928

**Published:** 2026-06-12

**Authors:** Haotian Zhang, Haowei Liu, Jiangpeng Xu, Xiaoling Li, Shasha Li, Xuemei Liu, Changhua Hu

**Affiliations:** 1 School of Pharmaceutical Sciences, Southwest University, Chongqing 400715, China; iamzht1995621@163.com (H.Z.);; 2 NMPA Key Laboratory for Quality Monitoring of Narcotic Drugs and Psychotropic Substances, Chongqing Institute for Food and Drug Control, Chongqing 401121, China

**Keywords:** cariprazine, D2/D3 receptor, glioblastoma, β-arrestin 2, drug repurposing

## Abstract

**Background:** Glioblastoma (GBM) is among the malignant tumors with the lowest five-year survival rate. Current treatments offer limited efficacy and first-line options are scarce, highlighting the urgent need for novel drugs. Cariprazine can cross the blood–brain barrier and has been reported to inhibit certain tumors; however, its effect on GBM remains unknown. This study aims to elucidate its anti-GBM effects and mechanisms. **Methods:** Cell proliferation and apoptosis were assessed by wound healing, Transwell, colony formation assays, flow cytometry and JC−10 staining. Co-immunoprecipitation (Co-IP) examined the effect of cariprazine on D2/D3–ARRB2 interaction. Direct binding of cariprazine to ARRB2 was determined by molecular docking and CETSA. Western blotting and immunofluorescence detected changes in proliferation and apoptosis-related proteins. In vivo anti-GBM activity was evaluated in subcutaneous mouse models. **Results:** Cariprazine inhibited GBM cell proliferation and migration, promoted apoptosis, and showed low astrocyte toxicity. In mice, it suppressed GBM allograft growth without overt systemic toxicity. These effects were mediated through D2/D3 receptors, as cariprazine disrupted the D2/D3–ARRB2 interaction and thereby inhibited ERK signaling. It also upregulated ARRB2, further inhibiting the growth of GBM. Molecular docking and CETSA confirmed the direct binding of cariprazine to ARRB2 at LEU-245 and PHE-246. **Conclusions:** This study is the first to repurpose cariprazine for GBM, elucidating a unique ARRB2-centered dual mechanism, thus offering a new therapeutic strategy.

## 1. Introduction

Glioblastoma (GBM) is a common primary brain tumor in adults, characterized by high invasiveness and an extremely poor prognosis [1,2]. Despite multiple treatment modalities, including surgical resection, radiotherapy, and temozolomide chemotherapy, the median survival of GBM patients is only approximately 12–15 months [3,4]. The poor clinical outcomes are primarily attributable to the intrinsic heterogeneity of GBM, its tumor-specific invasive growth patterns, and the rapid development of therapeutic resistance [5]. The blood–brain barrier (BBB) remains a major obstacle to effective treatment, as it prevents most systemic therapeutic agents from reaching the brain, thereby severely limiting therapeutic progress [6,7,8]. Consequently, developing novel strategies that can effectively cross the BBB represents one of the most pressing challenges in neuro-oncology.

In this context, drug repurposing holds considerable appeal. This strategy substantially reduces development time and costs while leveraging established clinical safety profiles, thereby accelerating clinical translation. Atypical antipsychotics (AAPDs) are particularly promising for GBM treatment because of their inherent ability to penetrate the BBB [9,10,11]. Epidemiological and preclinical evidence suggests that certain AAPDs possess antitumor activity. For example, fenfluridol inhibits tumor growth by suppressing AKT signaling [12], while thioridazine promotes autophagy-induced apoptosis via the Wnt/β-catenin pathway [13]. Notably, both drugs are dopamine D2-like receptor antagonists, suggesting that targeting dopaminergic signaling may be a viable therapeutic strategy for GBM.

The dopamine D2-like receptor family comprises three subtypes (D2, D3 and D4), which are highly expressed in the central nervous system [14]. D2 and D4 receptors are significantly upregulated in GBM tissue, and their expression levels serve as independent prognostic indicators of poor survival [15]. Functionally, gene silencing of D2 or D4 receptors significantly impairs the clonogenic capacity and survival of GBM cells [16,17]. In contrast, the role of the D3 receptor in GBM remains unclear. It is predominantly localized in brain regions such as the nucleus accumbens and raphe nuclei, which are less associated with motor side effects, suggesting a potentially favorable safety profile. However, recent evidence indicates that D3 receptor inhibitors suppress the growth of primary and temozolomide-resistant GBM cells, highlighting their potential therapeutic value [18].

**Figure 1:**
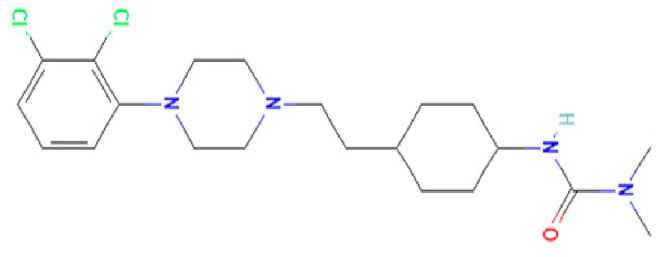
The 2D chemical structure of cariprazine. (Downloaded from pubchem).

Cariprazine (Figure 1) is a novel atypical antipsychotic that acts as a dopamine stabilizer, functioning as a partial agonist under low dopamine conditions and as an antagonist under high dopamine conditions [19,20,21]. Its unique pharmacological profile includes the highest D3 receptor binding affinity (Ki = 0.085 nM) among all antipsychotic drugs, as well as strong D2 receptor binding affinity (Ki_D2L_: 0.49 nM; Ki_D2S_: 0.69 nM) [22,23,24]. Functionally, cariprazine exhibits partial agonist activity at both D2 and D3 receptors, with EC50 values of approximately 1.9–5.8 nM and a maximal effect (Emax) of 45% for D3 receptors, and EC50 values of 0.18–3.2 nM with an Emax of approximately 30% for D2 receptors [25]. Compared with the full agonist quinpirole (Emax ≈ 100%), the intrinsic activity of cariprazine is markedly lower [25]. Haloperidol is a classic D2/D3 receptor antagonist. In a D3 antagonism functional assay, cariprazine (IC50 = 2.02 nM) and haloperidol (IC50 = 2.54 nM) demonstrated similar antagonistic efficacy [25]. Mechanistically, cariprazine displays biased signaling: it partially activates G protein-coupled pathways while acting as an antagonist of β-Arrestin 2 (ARRB2) recruitment, with an antagonistic potency comparable to that of the full antagonist haloperidol [26,27]. Recent preclinical studies have reported anticancer properties of cariprazine, including inhibition of HT29 colon cancer cell proliferation, sensitization of H460 non-small cell lung cancer cells to mitoxantrone [28,29], and anti-triple-negative breast cancer effects mediated through the promotion of autophagy, apoptosis, and immunomodulation [30].

However, whether cariprazine exerts therapeutic effects against GBM and the underlying molecular mechanisms remain unclear. Given the aberrant dopamine signaling in GBM, the high expression of D2 and D3 receptors, and cariprazine’s unique signaling bias and ability to cross the BBB, we hypothesize that cariprazine exerts anti-GBM effects by modulating the D2/D3 receptor–ARRB2 signaling axis.

This study aims to comprehensively validate the therapeutic efficacy of cariprazine against GBM in vitro and in vivo, and to elucidate the underlying mechanism of action, focusing on the D2/D3-ARRB2 signaling axis. Our findings provide a robust preclinical rationale for the use of cariprazine in GBM treatment and offer new insights into dopamine receptor signaling in cancer biology, potentially laying the groundwork for novel therapeutic strategies to overcome current challenges in GBM therapy.

## 2. Results

### 2.1. Cariprazine Inhibits GBM Cell Proliferation

**Figure 2:**
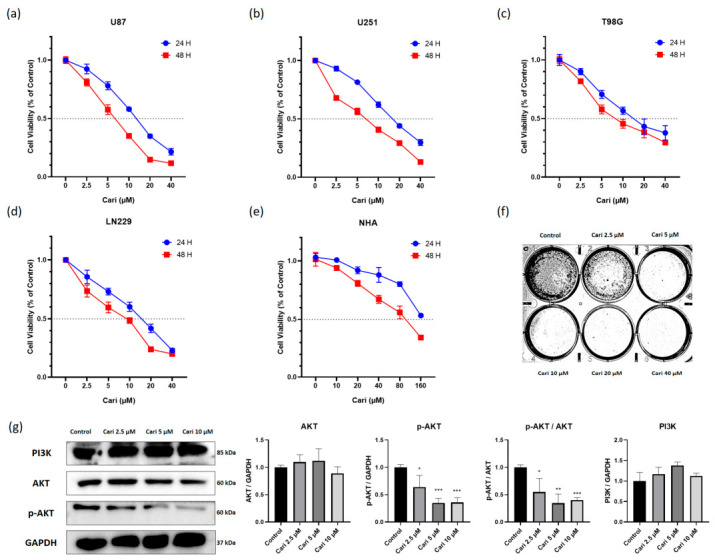
Cariprazine inhibits GBM cell proliferation. (**a**–**e**): Inhibitory ability of cariprazine on U87, U251, T98G, LN229, NHA cells; the dashed lines in the figure represented 50% cell viability; these results were expressed as mean ± SD, *n* = 5. (**f**): Different concentrations of cariprazine reduced the number of colonies in GBM cells within 15 days. (**g**): The effect of different concentrations of cariprazine on protein relative expression of PI3K-AKT pathway in GBM cells; these data were expressed as mean ± SD, *n* = 3; *: *p* < 0.05, **: *p* < 0.01, ***: *p* < 0.001 vs. control group.

To evaluate the inhibitory effect of cariprazine on GBM cell proliferation, the MTT assay was used to assess its impact on the viability of U87, U251, T98G, and LN229 cells. The IC50 values for each GBM cell line were as follows: U87 (IC50_24h_: 13.18 μM, IC50_48h_: 6.46 μM); U251 (IC50_24h_: 17.02 μM, IC50_48h_: 6.40 μM); T98G (IC50_24h_: 16.70 μM, IC50_48h_: 10.09 μM); LN229 (IC50_24h_: 14.02 μM, IC50_48h_: 7.84 μM) (Figure 2a–d). Cell viability decreased significantly with increasing cariprazine concentration. Colony formation assays further confirmed the inhibitory capacity of cariprazine against GBM cells (Figure 2f). Notably, the inhibitory effect of cariprazine on NHA astrocytes was weaker than that on GBM cells (IC50_24h_: 182.60 μM, IC50_48h_: 86.76 μM) (Figure 2e). Analysis of the PI3K-AKT pathway, a key regulator of GBM cell proliferation, revealed that cariprazine dose-dependently reduced p-AKT protein levels and the p-AKT/AKT ratio (Figure 2g). These results indicate that cariprazine effectively inhibits GBM cell proliferation while exerting a weaker effect on normal astrocytes.

### 2.2. Cariprazine Promotes Apoptosis in GBM Cells

**Figure 3:**
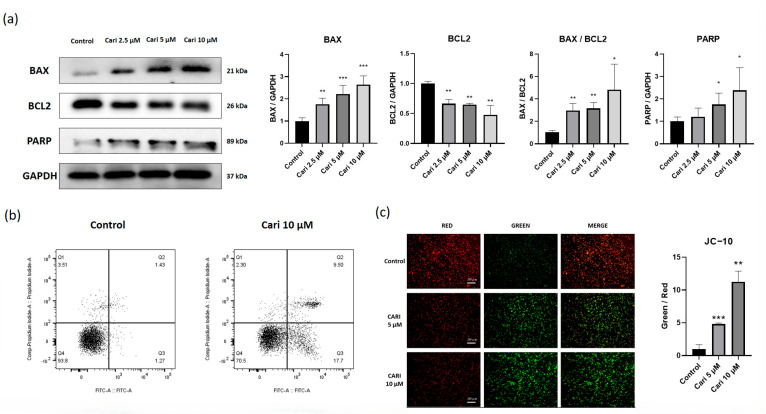
Cariprazine promotes GBM cell apoptosis. (**a**): The effect of different concentrations of cariprazine on protein relative expression of BAX, BCL2, PARP in GBM cells; these data were expressed as mean ± SD, *n* = 3; *: *p* < 0.05, **: *p* < 0.01, ***: *p* < 0.001 vs. control group. (**b**): Flow cytometry detection of the effect of cariprazine on apoptosis of GBM cells (Q1: Necrotic, Q2: Late Apoptosis, Q3: Early Apoptosis, Q4: Viable.). (**c**): JC−10 fluorescent probe detection of the effect of cariprazine on apoptosis of GBM cells.

The effects of cariprazine on GBM cell apoptosis were evaluated using Hoechst 33258 staining, JC−10 staining, and flow cytometry. Cariprazine dose-dependently increased Hoechst 33258 fluorescence intensity (Figure 3a) and the green/red fluorescence ratio of JC−10 (Figure 2d). Moreover, treatment with 10 μM cariprazine increased the percentage of cells in the Q2 quadrant (late apoptotic cells) in flow cytometry analysis (Figure 3c). Assessment of apoptosis-related proteins showed that cariprazine dose-dependently upregulated BAX and PARP protein levels as well as the BAX/BCL2 ratio, while downregulating the anti-apoptotic protein BCL2 (Figure 3b). These results demonstrate that cariprazine effectively promotes GBM cell apoptosis.

### 2.3. Cariprazine Inhibits GBM Cells Migration

**Figure 4:**
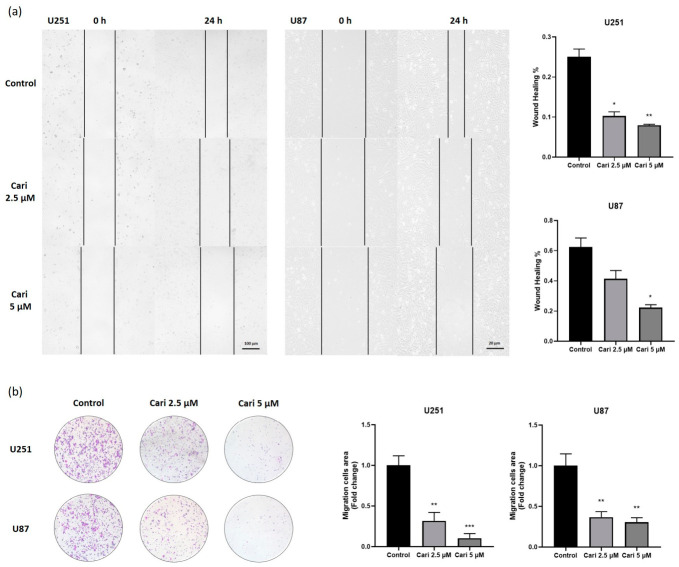
Cariprazine inhibits GBM cell migration. (**a**) Wound healing assay were conducted to investigate the effects of different concentrations of cariprazine on the migration of U87 and U251 cells. (**b**) Transwell experiment was conducted to investigate the effects of different concentrations of cariprazine on the migration of U87 and U251 cells. Data were expressed as mean ± SD, *n* = 3; *: *p* < 0.05, **: *p* < 0.01, ***: *p* < 0.001 vs. control group.

Given the highly migratory nature of GBM cells, we examined the effect of cariprazine on cell migration using wound healing and Transwell assays. In the wound healing assay, the control group exhibited significant wound closure after 24 h, whereas cariprazine dose-dependently inhibited wound closure (Figure 4a). The Transwell assay showed that a large number of control cells migrated through the membrane, whereas cariprazine significantly suppressed migration (Figure 4b). These findings indicate that cariprazine inhibits GBM cell migration.

### 2.4. Cariprazine Inhibits GBM Cells Growth In Vivo

**Figure 5:**
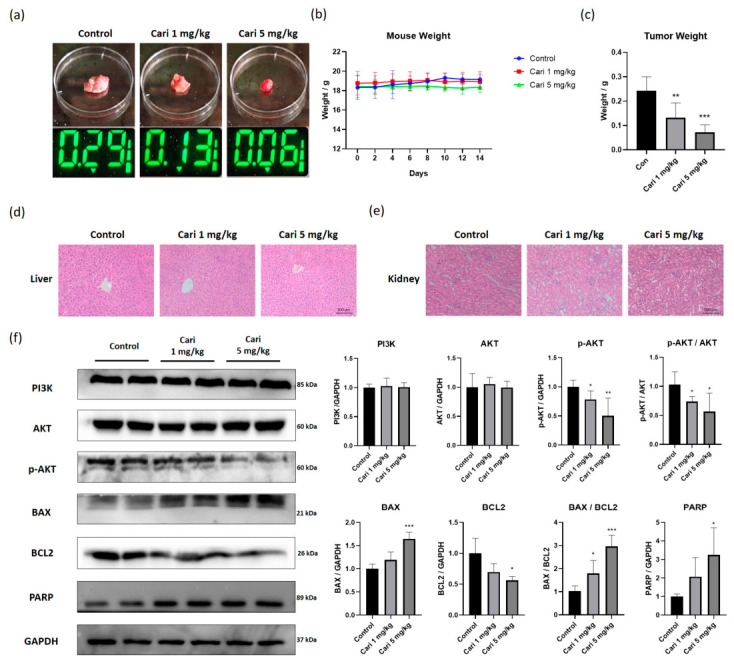
Cariprazine inhibits the growth of allograft tumors in mice. (**a**,**c**): Different concentrations of cariprazine reduced the weight of allograft tumors in mice. (**b**): The body weight of mice during cariprazine treatment. (**d**,**e**): H&E staining results of mouse liver and kidney. (**f**): The relative expression changes of PI3K-AKT and BAX, BCL2, PARP apoptosis proteins in mouse allograft tumors after cariprazine treatment. Data were expressed as mean ± SD, *n* = 3 for Western blot; *n* = 5 for animal experiment; *: *p* < 0.05, **: *p* < 0.01, ***: *p* < 0.001 vs. control group.

Based on the in vitro results showing that cariprazine inhibits proliferation and induces apoptosis, we next established a subcutaneous GBM allograft mouse model to evaluate its in vivo antitumor activity. After 14 days of treatment, cariprazine reduced subcutaneous tumor weight in a dose-dependent manner (Figure 5a,c), without significantly affecting mouse body weight (Figure 5b). H&E staining of the liver and kidney revealed no morphological alterations in these organs (Figure 5d,e), indicating that cariprazine at the therapeutic dose did not cause overt toxicity. Molecular analysis of tumor tissues showed that cariprazine did not affect total PI3K or AKT protein levels but significantly downregulated p-AKT and reduced the p-AKT/AKT ratio. Additionally, cariprazine upregulated the pro-apoptotic proteins BAX and PARP and increased the BAX/BCL2 ratio, while downregulating the anti-apoptotic protein BCL2 (Figure 5f). Collectively, these in vivo results demonstrate that cariprazine effectively suppresses GBM tumor growth without significant toxicity.

### 2.5. Cariprazine Exerts Its Anti-GBM Effect via D2/D3 Receptors

**Figure 6:**
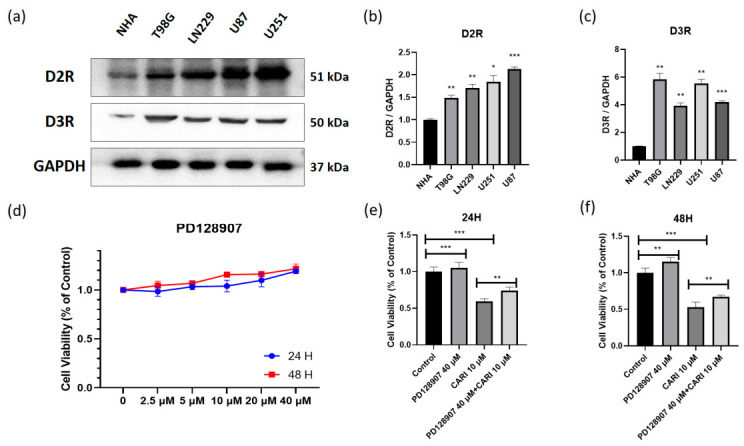
The anti-GBM effects of cariprazine are associated with D2/D3 receptors. (**a**–**c**): Relative expression levels of D2/D3 receptors in NHA, T98g, LN229, U87 and U251 cells. (**d**): The effects of PD128907 on the proliferation of GBM cells. (**e**,**f**): Cariprazine inhibited the proliferation of GBM cells after adding PD128907. Data were expressed as mean ± SD, *n* = 3 for Western blot; *n* = 5 for cell viability experiment; *: *p* < 0.05, **: *p* < 0.01, ***: *p* < 0.001.

**Figure 7:**
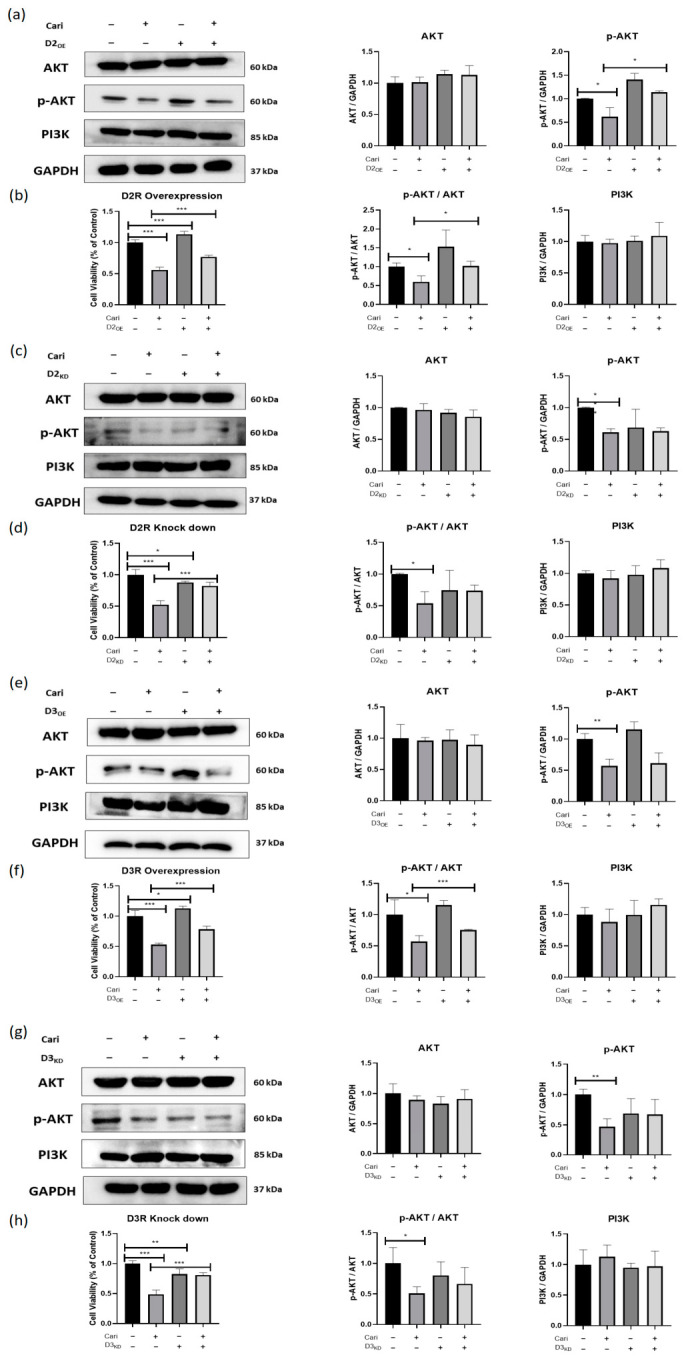
Cariprazine exerts antitumor effects through D2/D3 receptors. (**a**–**d**): The effect of overexpression or knockdown of D2 receptor on the proliferation activity of GBM cells and the expression of PI3K-AKT. (**e**–**h**): The effect of overexpression or knockdown of D3 receptor on the proliferation activity of GBM cells and the expression of PI3K-AKT. Data were expressed as mean ± SD, *n* = 3 for Western blot; *n* = 5 for cell viability experiment; *: *p* < 0.05, **: *p* < 0.01, ***: *p* < 0.001.

Given that cariprazine primarily targets D2/D3 receptors, we assessed their expression in GBM cells. D2 and D3 receptors were expressed at significantly higher levels in GBM cells than in NHA astrocytes; U251 cells exhibited the highest D2 expression, while T98G cells showed the highest D3 expression (Figure 6a–c). To determine whether D2/D3 receptors contribute to GBM growth, cells were treated with the D2/D3 receptor agonist PD128907. Activation of D2/D3 receptors significantly promoted GBM cell growth (Figure 6d), and this effect was markedly attenuated by cariprazine (Figure 6e,f), suggesting that GBM growth is mediated through D2/D3 receptors. We next established GBM cell models with D2/D3 receptor overexpression and knockdown. D2 receptor overexpression significantly promoted cell growth, and cariprazine inhibited this effect (Figure 7b) while reducing p-AKT levels and the p-AKT/AKT ratio (Figure 7a). Conversely, D2 receptor knockdown reduced cell proliferation and attenuated the inhibitory effect of cariprazine (Figure 7c,d). Similarly, D3 receptor overexpression increased cell growth, which was significantly suppressed by cariprazine (Figure 7f), accompanied by a decreased p-AKT/AKT ratio (Figure 7e). D3 receptor knockdown reduced proliferation and attenuated cariprazine’s inhibitory effect (Figure 7g,h). Taken together, these overexpression and knockdown experiments demonstrate that D2/D3 receptors are associated with GBM cell growth, and that cariprazine exerts its anti-GBM effects via these receptors.

### 2.6. ARRB2 Is a Key Target for the Anti-GBM Activity of Cariprazine

**Figure 8:**
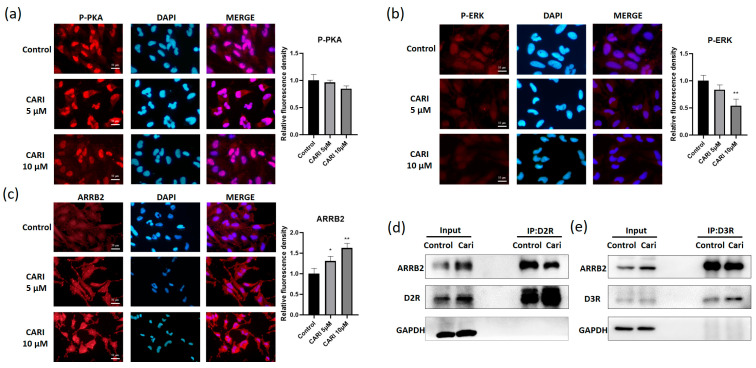
Cariprazine antagonizes the recruitment of ARRB2 by D2/D3 receptors. (**a**–**c**): Immunofluorescence detection of the effect of cariprazine on the protein expression of P-ERK, P-PKA, and ARRB2 in GBM cells. (**d**,**e**): The effect of cariprazine on D2 and D3 receptor coupled ARRB2. Data were expressed as mean ± SD; *: *p* < 0.05, **: *p* < 0.01.

**Figure 9:**
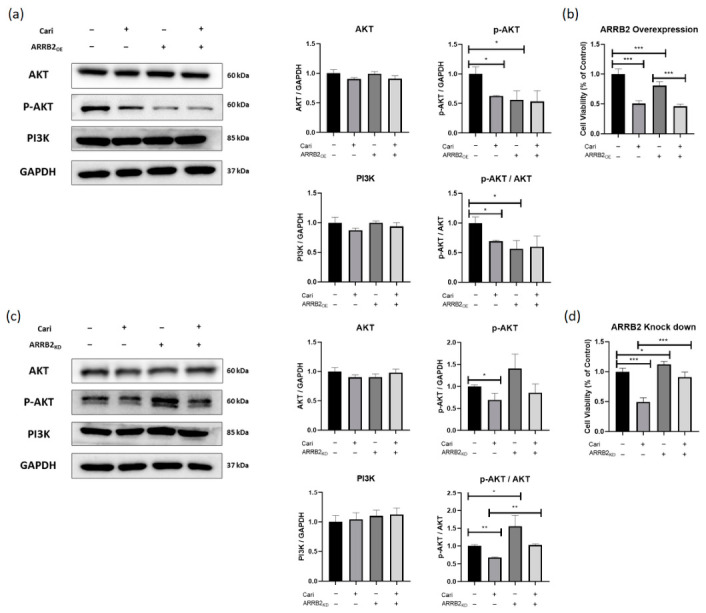
Cariprazine inhibits GBM cell proliferation through D2/D3 receptors coupled with ARRB2. (**a**,**b**): The effects of cariprazine on GBM cell proliferation and PI3K-AKT protein expression after overexpression of ARRB2. (**c**,**d**): The effects of cariprazine on GBM cell proliferation and PI3K-AKT protein expression after knock down of ARRB2. Data were expressed as mean ± SD, *n* = 3 for Western blot; *n* = 5 for cell viability experiment; *: *p* < 0.05, **: *p* < 0.01, ***: *p* < 0.001.

D2/D3 receptors signal primarily through Gi proteins and ARRB2. We assessed p-PKA (downstream of Gi) and p-ERK1/2 (downstream of ARRB2) by immunofluorescence. Cariprazine significantly reduced p-ERK1/2 levels (Figure 8b) but only weakly affected p-PKA (Figure 8a), suggesting that cariprazine weakly activates the Gi pathway while strongly antagonizing ARRB2-mediated signaling. We performed CO-IP experiments using D2 and D3 receptors as bait proteins. In the input group, protein levels of D2 receptors and ARRB2 increased following cariprazine treatment, whereas in the IP group, levels of D2 and D3 receptors increased after cariprazine treatment, while ARRB2 levels decreased. These results further demonstrate that cariprazine antagonizes the recruitment of ARRB2 by D2/D3 receptors (Figure 8d,e). These results point to ARRB2 as a key mediator of cariprazine’s anti-GBM activity. In addition, immunofluorescence analysis revealed that cariprazine treatment significantly increased ARRB2 protein levels (Figure 8c). We therefore established ARRB2-overexpressing and ARRB2-knockdown GBM cell models to further investigate its role. ARRB2 overexpression significantly inhibited GBM cell growth and reduced both p-AKT levels and the p-AKT/AKT ratio (Figure 9a,b). In contrast, ARRB2 knockdown markedly increased cell growth and elevated the p-AKT/AKT ratio; the anti-proliferative effect of cariprazine was also attenuated under these conditions (Figure 9c,d). Collectively, these results indicate that cariprazine inhibits GBM growth both by antagonizing the D2/D3-ARRB2-ERK pathway and by upregulating ARRB2, identifying ARRB2 as a key target in its anti-GBM mechanism.

### 2.7. Cariprazine Binds Directly to ARRB2

**Figure 10:**
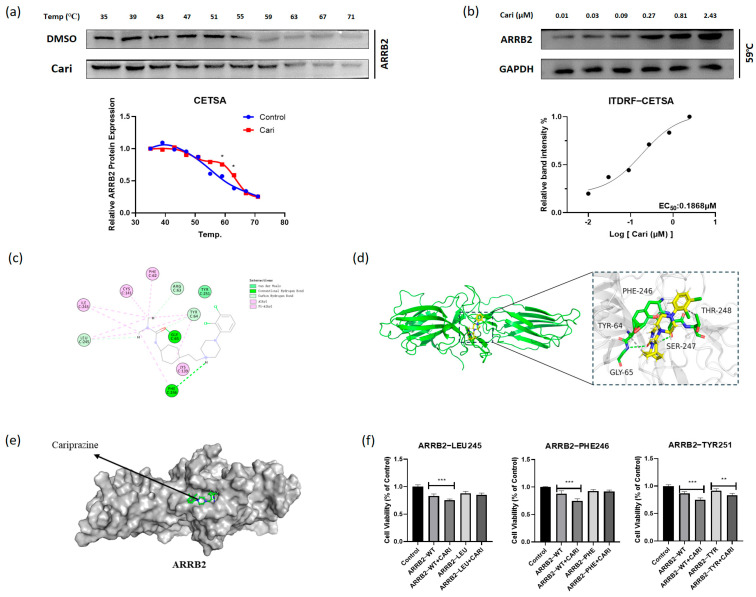
Prediction of cariprazine binding to ARRB2. (**a**): CETSA experiment detected the binding of cariprazine and ARRB2. (**b**): ITDRF–CETSA experiment detected the binding effect of cariprazine and ARRB2. (**c**): The binding of amino acid of ARRB2 to cariprazine. (**d**,**e**): 3D structural simulation diagram of cariprazine bound with ARRB2. (**f**): The effect of cariprazine on ARRB2–245LEU, ARRB2–246PHE, ARRB2–251TYR mutant GBM cells. Data were expressed as mean ± SD, *n* = 5 for cell viability experiment; *: *p* < 0.05, **: *p* < 0.01, ***: *p* < 0.001.

Molecular docking analysis showed that cariprazine binds to ARRB2, with potential interaction sites located within the GPCR-interacting finger domains of ARRB2 (Figure 10d,e). Leu245, Phe246, and Tyr251 were predicted to be the primary binding residues (Figure 10c). The CETSA confirmed that cariprazine significantly protected ARRB2 from thermal degradation at 59 °C and 63 °C (Figure 10a). The ITDRF–CETSA experiment further confirmed that cariprazine has dose-dependent binding to ARRB2 protein, and its EC50 is approximately 0.1868 μM (Figure 10b). Point mutation analysis further demonstrated that mutations at Leu245 and Phe246 significantly attenuated the anti-proliferative effect of cariprazine in GBM cells, whereas mutation at Tyr251 had virtually no effect (Figure 10f). These results identify Leu245 and Phe246 as key binding residues through which cariprazine may disrupt the recruitment of ARRB2 by D2/D3 receptors.

## 3. Discussion

The clinical treatment landscape for GBM remains challenging, with five-year survival rates remaining low [31]. The BBB significantly impedes the efficiency of drug development. Drug repurposing represents a cost-effective strategy that can substantially reduce both the time and cost associated with drug development [32,33]. Notably, AAPDs are promising candidate agents for anti-GBM therapy because of their ability to cross the BBB [10,11]. This study elucidates the role and mechanism of cariprazine in anti-GBM therapy and demonstrates that cariprazine significantly inhibits GBM cells, primarily through the D2/D3–ARRB2 signaling axis.

Cariprazine is a third-generation antipsychotic characterized by high affinity for the D3 receptor; its partial agonism at D2/3 receptors contributes to a reduced incidence of adverse effects. This study demonstrates that cariprazine inhibits the PI3K/AKT signaling pathway in GBM cells, thereby exerting anti-proliferative effects. It also promotes apoptosis by upregulating pro-apoptotic factors such as PARP and BAX, while downregulating BCL2. These findings were corroborated in vivo. In mouse allograft models, cariprazine at various concentrations significantly inhibited the growth of subcutaneous GBM tumors, with no significant hepatotoxicity or nephrotoxicity observed. The anti-proliferative and pro-apoptotic effects of cariprazine have also been reported in other cancer types. For instance, in breast cancer (MDA-MB-231), colorectal cancer (HCT116), and cervical cancer (HeLa) cells [34], cariprazine inhibited proliferation by suppressing p-AKT and increased the expression of BAX and caspase-3 to promote apoptosis. These findings suggest that cariprazine possesses considerable anticancer activity with a favorable safety profile.

The primary innovation of this study is the discovery of a novel anticancer mechanism of cariprazine, which operates through dopamine D2/3 receptors that are highly expressed in GBM. Dopamine signaling is highly active in GBM; activation of D1 receptors or antagonism of D2 receptors exerts anti-GBM effects, whereas elevated dopamine levels paradoxically promote tumor growth [35,36]. Consistent with the results of this study, most D2 receptor antagonists have demonstrated significant antitumor effects in various cancers, including GBM [37,38], cervical cancer [34], prostate cancer [39], and colorectal cancer [34]. Here, treatment of GBM cells with the D2/3 agonist PD128907 confirmed that activation of D2/3 receptors promotes GBM proliferation. Furthermore, through overexpression and knockdown of D2/3 receptors, it was demonstrated that cariprazine inhibits GBM proliferation mainly via D2/3 receptors. D2/3 receptors transmit signals primarily through Gi protein and ARRB2 [40]. The results showed that cariprazine had a weaker effect on PKA signaling downstream of Gi protein than on ERK signaling downstream of ARRB2. Cariprazine significantly inhibited ERK, indicating biased modulation of D2/D3 receptors. In addition, this study found that cariprazine inhibits the recruitment of ARRB2 by D2/3 receptors. Previous studies have also confirmed that cariprazine antagonizes ARRB2 recruitment to an extent comparable to the full antagonist haloperidol [26], consistent with our findings. Together, these results demonstrate that cariprazine exerts anti-GBM effects by inhibiting the D2/3–ARRB2 axis.

Interestingly, this study also uncovered an additional role for ARRB2. Cariprazine upregulated the overall expression level of ARRB2. Additional investigations revealed that the inhibitory effect of cariprazine was further enhanced in GBM cells overexpressing ARRB2, whereas knockdown of ARRB2 attenuated its anti-GBM efficacy. However, we found that the effects of ARRB2 overexpression and knockdown were relatively modest in this study. Our results show that overexpression of ARRB2 in GBM cells significantly reduced cell viability by approximately 19.4%, whereas ARRB2 knockdown significantly increased cell viability by approximately 12.3%. Although the proliferative effect was not particularly strong, the inhibitory effect of cariprazine was reduced from approximately 50% to 10%, indicating that the anti-GBM activity of cariprazine is ARRB2-dependent. If other proteins were primarily responsible for mediating this effect, we would have observed that the drug retained a significant degree of inhibitory activity following ARRB2 knock down; however, this was not the case. In addition, the relatively short treatment duration may also account for this modest effect. In this study, the treatment lasted only 48 h, so the full effects of ARRB2 may not have been completely realized. According to related studies on ARRB2 in other cancer types, glioblastoma, non-small cell lung cancer, and bladder cancer cells are all relatively sensitive to ARRB2. For example, following two weeks of ARRB2 overexpression in the GBM cell line U87, colony number decreased by approximately 50% [41]. In the bladder cancer cell line UM-UC-3, ARRB2 knockout led to a marked increase in cell invasiveness, with colony number increasing by approximately 70–150% [42]. In the lung cancer cell lines H1299 and A549, ARRB2 knockout also resulted in a significant increase in colony formation. Moreover, in H1299 cells, cell viability showed no significant difference on days 1–2, but a relatively pronounced proliferative effect emerged on day 3. In A549 cells, no significant differences in cell viability were observed on days 1–3, but a relatively pronounced proliferative effect appeared on day 5 [43]. These findings suggest that the effect of ARRB2 may develop relatively slowly and gradually increase over time. Although the effects of ARRB2 overexpression and knockdown on GBM cells are modest at the basic level, ARRB2 is critical for the antitumor activity of cariprazine against GBM. Thus, we identify ARRB2 as a key target that mediates the anti-GBM effect of cariprazine.

Moreover, this study revealed that cariprazine can bind directly to the ARRB2 protein. Previous work by Yin et al. demonstrated that the ridge region of ARRB2 interacts with the intracellular loop 1 (ICL1) domain of GPCRs [44]. Using molecular docking and CETSA, this study found that cariprazine binds to the ridge region of ARRB2. Site-directed mutagenesis experiments further identified Leu245 and Phe246 as the primary binding sites. This suggests that cariprazine may inhibit the recruitment of ARRB2 by D2/3 receptors by directly binding to ARRB2, thereby exerting anti-GBM effects.

This study elucidates the anti-GBM effects and mechanisms of cariprazine, although certain limitations remain. First, the findings are based primarily on preclinical models, including in vitro experiments and in vivo mouse allograft transplantation studies. Future clinical studies are needed to evaluate the therapeutic potential of cariprazine and other AAPDs in patients with GBM. Additionally, given that ARRB2 expression varies across different cancers, whether cariprazine exerts antitumor effects through similar ARRB2-dependent mechanisms in other cancer types warrants further investigation.

## 4. Materials and Methods

### 4.1. Cell Culture and Reagents

The human GBM cell lines U251, U87, LN229, T98G and NHA were provided by the laboratory of Professor Hongjuan Cui. The mouse GBM cell lines GL261 were purchased from the National Centre for Tissue Culture Collection (Shanghai, China). All cell lines were maintained in Dulbecco’s Modified Eagle’s Medium (DMEM; Gibco, Waltham, MA, USA) supplemented with 10% fetal bovine serum (FBS; Adamas Life, Shanghai, China) and cultured at 37 °C in a humidified atmosphere containing 5% CO_2_. All cell lines used in this study were confirmed to be mycoplasma-free.

### 4.2. Plasmid Construction and Transfection

For gene overexpression, the open reading frames (ORFs) of DRD2, DRD3 and ARRB2 were amplified by PCR and cloned into the pCDH-EF1a-CMV-Puro vector. For gene silencing, the commercial plasmid pX459 was used. All constructs were validated by Sanger sequencing. Site-directed mutagenesis was performed on ARRB2 using specific primers to generate point mutants. Restriction enzymes were purchased from TAKARA (Kusatsu, Japan) and New England Biolabs (Ipswich, MA, USA), whilst DNA ligase was sourced from Vazyme Biotech (Nanjing, China). When cells reached 70–80% confluence, transient transfection was performed using Lipo8000 transfection reagent according to the manufacturer’s protocol. Six hours after transfection, the medium was replaced with fresh medium, and puromycin selection was carried out prior to subsequent experiments.

### 4.3. Drug Preparation and Treatment

Cariprazine (Aladdin, Shanghai, China, C171931) and PD128907 (Aladdin, Shanghai, China, P129442) were dissolved in dimethyl sulphoxide (DMSO) to prepare 10 mM stock solutions, which were sonicated to ensure complete dissolution. Working solutions were freshly diluted in culture medium to final concentrations of 2.5, 5, 10, 20, and 40 μM. For in vivo administration, cariprazine was suspended in 0.5% sodium carboxymethylcellulose (CMC-Na) at a concentration of 2 mg/mL. For animal treatment, cariprazine was administered orally once daily.

### 4.4. Cell Viability Assay

Cell survival rates were assessed using the MTT [3-(4,5-dimethylthiazol-2-yl)-2,5-diphenyl tetrazolium bromide] assay. Cells were seeded into 96-well plates at a density of 3000 cells per well. After 24 h, the cells were treated with the specified compound. At 24 and 48 h post-treatment, 20 μL of MTT solution (5 mg/mL; Meilun Bio, Dalian, China, MB4698-1) was added to each well and incubated for 4 h. The crystals of metazamide were dissolved in DMSO, and the absorbance was measured at a wavelength of 490 nm.

### 4.5. Clonogenic Assay

Cells were seeded at a low density of 1000 cells per well in a 6-well plate and cultured for 72 h to form colonies. Subsequently, cells were treated with different concentrations of cariprazine for 48 h, followed by 10 days of culture in standard medium. The resulting cell colonies were then fixed with 4% paraformaldehyde and stained with 0.1% crystal violet solution. After gentle washing with phosphate-buffered saline (PBS), the stained colonies were photographed and quantified.

### 4.6. Wound Healing Assay

Cells were seeded at a density of 5 × 10^5^ cells per well in a 6-well plate and allowed to grow until confluence reached approximately 80–90%. A uniform wound was created in the cell monolayer using a sterile 200 μL pipette tip. After washing to remove detached cells, fresh medium containing the specified treatment was added. Images of the wound area were captured at 0 and 24 h using an inverted microscope to monitor cell migration.

### 4.7. Transwell Assay

500 μL of culture medium supplemented with 10% serum were added to the lower chamber of a 24-well plate. Then, 5 × 10^4^ GBM cells were seeded into the upper chamber of the Transwell insert and incubated for 48 h. At the end of the incubation period, cells that had migrated to the underside of the Transwell membrane were fixed. The fixed cells were subsequently stained with crystal violet. Finally, the area occupied by the stained cells was quantified.

### 4.8. Cell Apoptosis Analysis

Apoptosis was assessed by Annexin V-FITC/propidium iodide (PI) double staining (Mei5bio, Beijing, China, MF123) followed by flow cytometry. Cells were stained with Annexin V-FITC and PI for 15 min at room temperature in the dark. Stained cells were analyzed within 1 h using a flow cytometer. Early apoptotic cells were defined as Annexin V^+^/PI^−^, late apoptotic cells as Annexin V^+^/PI^+^, and necrotic cells as Annexin V^−^/PI^+^. Mitochondrial membrane potential was monitored using the JC–10 fluorescent probe (Beyotime, Shanghai, China, CDX-T0103-M001) in accordance with the manufacturer’s instructions.

### 4.9. Immunofluorescence

Following drug treatment, cells were fixed with 4% paraformaldehyde, permeabilized with 0.1% Triton X-100, and blocked with 1% bovine serum albumin (BSA). The cells were then incubated overnight at 4 °C with ARRB2 antibody (Solarbio, Beijing, China, K002346P), P-PKA antibody (Santa Cruz, CA, USA, sc-293036) and p-ERK1/2 antibody (Abclonal, Wuhan, China, AP0974) at a dilution of 1:500. After washing, the cells were incubated with Alexa Fluor 488-conjugated secondary antibody (Beyotime, Shanghai, China, A0423). The cell nuclei were counterstained with DAPI, and images were acquired using a fluorescence microscope.

### 4.10. Co-Immunoprecipitation (Co-IP)

The cells were washed with cold PBS and lysed using IP lysis buffer. The cell lysates were centrifuged to collect the supernatant, which was then gently stirred overnight at 4 °C with antibody-conjugated magnetic beads (Bio-Rad, Hercules, CA, USA). The immune complexes bound to the beads were collected, eluted by heating in SDS loading buffer, and subsequently analyzed by Western blotting.

### 4.11. Animal Studies

Prior to the experiment, eight-week-old female C57BL/6 mice were acclimatized for one week. Each mouse was inoculated with 1 × 10^7^ GL261 cells near the right axilla. Drug treatment was initiated one week after tumor implantation and continued for 14 days. Mice were euthanized, and tumors and major organs were harvested for subsequent analysis. Animal experiments were conducted in accordance with relevant guidelines and regulations and were approved by the Institutional Animal Care and Use Committee of Southwest University (Approval No.: IACUC-20250506-02).

### 4.12. H&E Staining

Tissue sections were fixed in 4% paraformaldehyde, embedded in paraffin, and sectioned into 4 μm thick slices. The tissue sections were dewaxed and rehydrated according to standard protocols, and stained with hematoxylin and eosin (H&E). The stained sections were examined and imaged under a light microscope.

### 4.13. Cell Heat Shift Assay (CETSA)

Cells were treated with cariprazine or DMSO for 4 h. Cell lysates were collected, divided into 10 equal portions, and heated for 3 min at different temperatures. The heated samples were flash-frozen in liquid nitrogen, thawed at room temperature, centrifuged to collect the supernatant, and finally analyzed by Western blot.

For the isothermal dose–response fingerprint CETSA experiment, cells were treated with different concentrations of cariprazine (0.01 μM, 0.03 μM, 0.09 μM, 0.27 μM, 0.81 μM, 2.43 μM) for 4 h. Cell lysates were collected, heated at 59 °C for 3 min, then rapidly frozen in liquid nitrogen. After thawing at room temperature and centrifugation, the supernatant was collected and analyzed by Western blot.

### 4.14. Molecular Docking

The 3D chemical structure of cariprazine was retrieved from the PubChem database. The crystal structure of ARRB2 (PDB ID: 8J9K) was obtained from the Protein Data Bank. Molecular docking simulations were performed using the LibDock module in Discovery Studio software (v19.1.0). The docking results were visualized and analyzed using PyMOL molecular visualization software (v2.5.4).

### 4.15. Western Blotting

Proteins were extracted from cells and tissues (tissues required homogenization) using RIPA lysis buffer containing PMSF and phosphatase inhibitors (Beyotime, Shanghai, China). After 30 min of lysis on ice, the supernatant was collected by centrifugation. Total protein concentration was determined using a BCA protein quantification kit (Servicebio, Wuhan, China). An SDS-PAGE gel was prepared for protein separation, and the proteins on the gel were then transferred to a PVDF membrane (Merck, Darmstadt, Germany). Following a 1 h block with 5% skimmed milk powder, the primary antibody was incubated overnight. Incubate with a secondary antibody matching the source of the primary antibody for 1.5 h, then add ECL development solution (Wanlei Bio, Shenyang, China) and place in an imaging system (Clinx, Shanghai, China) for exposure and photography. Image greyscale analysis was performed using ImageJ software (v1.53t). The primary antibodies used were as follows: GAPDH (Servicebio, Wuhan, China, GB11002), PI3K (Wanlei Bio, Shenyang, China, WL02240), AKT (Wanlei Bio, Shenyang, China, WL0003b), P-AKT Ser473 (Wanlei Bio, Shenyang, China, WLP001a), BCL2 (Huabio, Hangzhou, China, HA721235), BAX (Huabio, Hangzhou, China, ER0907), P-ERK1/2 (Abclonal, Wuhan, China, AP0974), PARP (Huabio, Hangzhou, China, ET1608-10), DRD2 (Wanlei Bio, Shenyang, China, WL01122), DRD3 (Huabio, Hangzhou, China, HA721391), ARRB2 (Solarbio, Beijing, China, K002346P). The secondary antibodies used are as follows: HRP-conjugated goat anti-rabbit IgG (ServiceBio, Wuhan, China, GB23303), HRP-conjugated goat anti-mouse IgG (ServiceBio, Wuhan, China, GB23301).

### 4.16. Statistical Analysis

Statistical analysis was performed using SPSS 20.0 software, and graphs were generated using GraphPad Prism 8. Data are expressed as mean ± standard deviation (mean ± SD). In accordance with standard practice, each experimental group included at least three biological replicates; for animal studies adhering to the ‘3Rs’ principles, each group comprised six animals as replicates. Multi-group comparisons were performed using one-way analysis of variance (ANOVA) with LSD multiple comparison tests, whilst comparisons between two groups were performed using two-tailed unpaired Student’s *t*-tests. A *p*-value of <0.05 was considered statistically significant.

## 5. Conclusions

This study demonstrates that the atypical antipsychotic cariprazine exhibits potent anti-GBM activity in both in vitro and in vivo models. Mechanistic analyses of dopamine D2/D3 receptors and their coupling partner ARRB2 reveal that cariprazine exerts anti-GBM effects by inhibiting D2/D3 receptor-mediated ARRB2 recruitment. Furthermore, cariprazine suppresses GBM progression through ARRB2-dependent pathways, confirming ARRB2 as a critical therapeutic target. Collectively, this work establishes a preclinical foundation for repurposing AAPDs in GBM treatment and provides robust experimental evidence supporting the potential of cariprazine as a novel anti-GBM agent.

## Data Availability

The original contributions presented in this study are included in the article. Further inquiries can be directed to the corresponding authors.

## Abbreviations

The following abbreviations are used in this manuscript:

GBM	Glioblastoma
Co-IP	Co-immunoprecipitation
CETSA	Cell Heat Shift Assay
ITDRF–CETSA	isothermal dose–response fingerprint CETSA
BBB	blood–brain barrier
AAPDs	Atypical antipsychotics
ARRB2	β-Arrestin 2
ORFs	open reading frames
DMSO	dimethyl sulphoxide
CMC-Na	carboxymethylcellulose
PI	propidium iodide
